# Ni-CNT Chemical Sensor for SF_6_ Decomposition Components Detection: A Combined Experimental and Theoretical Study

**DOI:** 10.3390/s18103493

**Published:** 2018-10-16

**Authors:** Yingang Gui, Xiaoxing Zhang, Peigeng Lv, Shan Wang, Chao Tang, Qu Zhou

**Affiliations:** 1 College of Engineering and Technology, Southwest University, Chongqing 400715, China; tangchao_1981@163.com (C.T.); zhouqu@swu.edu.cn (Q.Z.); 2 State Grid Chongqing Shiqu Power Supply Company, Chongqing 400015, China; 13983772950@139.com (P.L.); shan102330@msn.com (S.W.); 3 School of Electrical Engineering, Wuhan University, Wuhan 430072, China

**Keywords:** SF_6_ decomposition components, carbon nanotube sensor, Ni modification, adsorption, DFT calculations

## Abstract

SF_6_ decomposition components detection is a key technology to evaluate and diagnose the insulation status of SF_6_-insulated equipment online, especially when insulation defects-induced discharge occurs in equipment. In order to detect the type and concentration of SF_6_ decomposition components, a Ni-modified carbon nanotube (Ni-CNT) gas sensor has been prepared to analyze its gas sensitivity and selectivity to SF_6_ decomposition components based on an experimental and density functional theory (DFT) theoretical study. Experimental results show that a Ni-CNT gas sensor presents an outstanding gas sensing property according to the significant change of conductivity during the gas molecule adsorption. The conductivity increases in the following order: H_2_S > SOF_2_ > SO_2_ > SO_2_F_2_. The limit of detection of the Ni-CNT gas sensor reaches 1 ppm. In addition, the excellent recovery property of the Ni-CNT gas sensor makes it easy to be widely used. A DFT theoretical study was applied to analyze the influence mechanism of Ni modification on SF_6_ decomposition components detection. In summary, the Ni-CNT gas sensor prepared in this study can be an effective way to evaluate and diagnose the insulation status of SF_6_-insulated equipment online.

## 1. Introduction

Due to the strong insulation strength and electronegativity of SF_6_ gas, it has been widely used as filling gas in insulated equipment, such as SF_6_-insulated high-voltage switchgear (GIS), SF_6_-insulated transmission lines (GIL), and SF_6_-insulated circuit breakers (GCB) [1,2,3]. However, insulation defects inevitably occur in SF_6_-insulated equipment, which induces SF_6_ to decompose into various decomposition components: SO_2_, H_2_S, SOF_2_, and SO_2_F_2_ under electric discharge [4,5,6,7]. In addition, the existence of insulation defects and SF_6_ decomposition dramatically reduce the insulation strength of SF_6_-insulated equipment [8]. A lot of methods have been studied to detect the insulation defects, including the ultra-high frequency method (UHF) [9,10], the transient earth voltage method (TEV) [11], the ultrasonic method [12],the gas chromatographic method [13], and the gas sensor detection method [14]. However, UHF, TEV, and the ultrasonic method are easily affected by interference signals, and the gas chromatographic method is an offline detection method. Because of the non-contact, high accuracy, and low detection limit features of gas sensors, the gas sensor detection method has been an effective way to realize the online detection of SF_6_-insulated equipment based on SF_6_ decomposition components detection [15,16].

One dimensional carbon nanotube (CNT) material has attracted extensive study due to its outstanding characteristics [17,18], such as large specific surface area and good electrical properties. It is demonstrated that CNTs shows excellent gas sensing performance to gases [19,20], making it an effective detection method used in environmental, industrial, and military fields [21,22]. Li et al. fabricated single-wall carbon nanotube (SWNT) based gas sensors on interdigitated gold electrodes; its detection limit reaches 44 ppb for NO_2_ and 262 ppb for nitrotoluene at room temperature [19]. Chopra et al. reported that multi-wall carbon nanotube (MWCNT) and polymer composites exhibited excellent sensitivity and selectivity to NH_3_ and NO_2_ at room temperature [23]. Surface modification based on metal particles can greatly improve the gas sensitivity and selectivity of CNTs. Penza et al. verified that the functionalization of Pt and Pd nanoclusters on the MWCNT surface significantly improved its detection limit to sub-ppm level upon NO_2_, H_2_S, NH_3_, and CO detection [24]. Although the development of CNT gas sensors has made a remarkable improvement in common gas detection, there are few reports about its application in the field of SF_6_ decomposition components detection.

Here, Nickel functionalized CNTs (Ni-CNTs) are adopted as the gas sensing material to detect the characteristic SF_6_ decomposition components: SO_2_, H_2_S, SOF_2_ and SO_2_F_2_. Experimentally, the Ni-CNT gas sensor is prepared by the drop-casting method, which significantly reduces the preparation cost and benefits for industrial-scale production. In addition, the gas sensing mechanism of Ni-CNTs to the characteristic SF_6_ decomposition components is studied by density functional theory (DFT) calculation. We conclude that the Ni-CNT gas sensor can be an effective way to detect the insulation defects and diagnose the running status of SF_6_-insulated equipment online.

## 2. Material and Methods

### 2.1. Synthesis of Ni-CNT Gas Sensor

**Figure 1:**
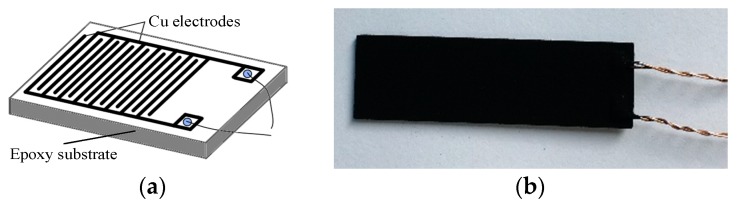
(**a**) Interdigitated Cu electrodes, (**b**) the prepared Ni-modified carbon nanotube (Ni-CNT) gas sensor.

Ni-CNTs (inner diameter: 5–10 nm, external diameter: 10–20 nm, and length: 10–30 μm) were bought from Aladdin Reagent Corporation (Shanghai, China). Figure 1a shows the interdigitated Cu electrodes; Cu electrodes (thickness: 30 μm, gap: 0.2 mm) were fabricated on epoxy resin as a substrate of the Ni-CNT gas sensor. Ni-CNTs were dispersed in dimethylformamide (DMF) to form a solution of 0.025 mg/mL by the following steps: soak 6 h, then ultrasonic treat 2 h. The solution was sprayed onto the surface of the Cu electrodes at 80 °C to accelerate the evaporation of DMF. As shown in Figure 1b, Ni-CNTs evenly distribute on the surface of the Cu electrodes, which benefits the gas molecule adsorption and desorption process.

### 2.2. Material Characterization and Gas Sensing Measurement

Transmission electron microscopy (TEM) was tested by FEI Tecnai G2 F20 S-TWIN at 200 kV. The gas-sensing detection system was composed of standard gases (SO_2_, H_2_S, SOF_2_, and SO_2_F_2_), the gas distribution system, the gas sensing chamber, and an electrochemical workstation. The concentration of standard gases was 400 ppm with N_2_ as the background gas. The standard gases were diluted to different concentrations: 1, 10, 50, 100, 200, 300, and 400 ppm by the gas distribution system. Then, the diluted gases were led to the closed gas chamber. The resistance of the Ni-CNT gas sensor was measured by the electrochemical workstation at room temperature and ambient pressure. The gas response (*GR*) was defined by Equation (1). In which, *R_i_* is the initial electric resistance of the Ni-CNT gas sensor in dry synthesis air and *R_f_* is the resistance of the sensor in SF_6_ decomposition components’ atmosphere. In addition, the sensitivity (*S*) of the Ni-CNT gas sensor was defined by Equation (2), where *C_T_* was the concentration of tested gas.

(1) $$GR=\frac{R_{f}-R_{i}}{R_{i}}\times 100\%$$

(2) $$S=\frac{G.R}{C_{T}}\times 100\%$$

### 2.3. DFT Calculation

The DFT calculations were performed using Dmol^3^ package of materials studio. The structure of (8, 0) Ni-CNTs was built by substituting a carbon atom by one Ni atom. In addition, a 20 × 20 × 8.5 Å periodic-boundary supercell was built to avoid the interference of adjacent cells. Then, the optimized adsorption structures of SO_2_, H_2_S, SOF_2_, and SO_2_F_2_ on the surface of Ni-CNTs were calculated with different initial gas positions. The Perdew–Burke–Ernzerhof (PBE) function generalized gradient approximation was adopted to treat the exchange-correlation potential. The convergence criterion for energy and force was set at 10^−5^ Ha and 2 × 10^−3^ Ha/Å, respectively. The Brillouin zone was sampled with 1 × 1 × 2 Monkhorst–Pack mesh. The adsorption energy (*E_ads_*) of the molecules adsorbed on Ni-CNTs was defined by the following Equation (3):

(3) $$E_{ads}=E_{gas/surf}-E_{gas}-E_{surf}$$

where *E_gas/suf_* was the energy of gas adsorbed CNTs, *E_surf_* and *E_gas_* were the energy of isolated Ni-CNTs and molecules of SF_6_ decomposition components.

The electron density distribution was obtained by Mulliken population analysis. The charge transfer (*Q*_t_) in the adsorption process was defined by Equation (4), where *Q_iso_* and *Q_ads_* were the respective total charge of gas molecules before and after adsorption.

(4) $$Q_{t}=Q_{ads}-Q_{iso}$$

## 3. Results and Discussion

### 3.1. Preparation and Gas Sensing Property of Ni-CNT Gas Sensor

**Figure 2:**
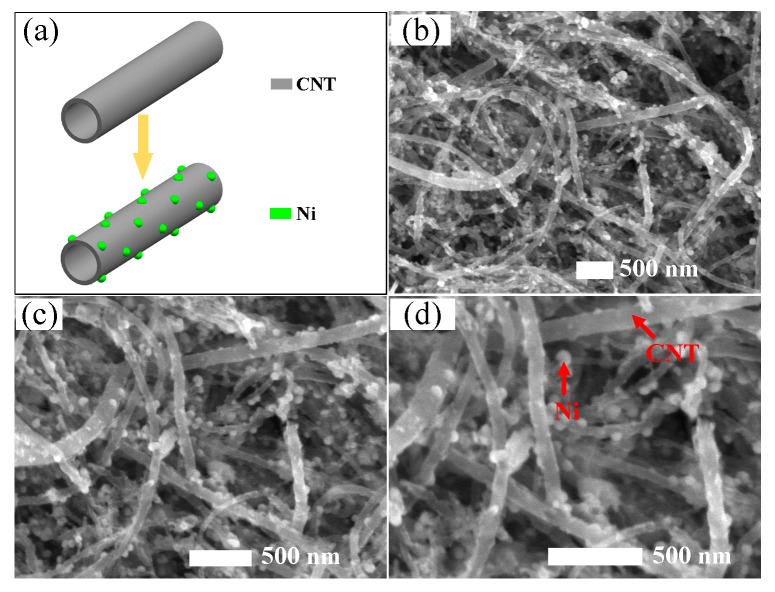
(**a**) The formation mechanism of Ni-CNTs, (**b**–**d**) structure characterization of Ni-CNTs.

As per the formation mechanism of Ni-CNTs shown in Figure 2a, it is important to make sure that Ni particles evenly decorate onto the outside wall of intrinsic CNTs, which plays a key role to the gas-sensing properties of the prepared Ni-CNT gas sensor. As per the typical TEM images shown in Figure 2b–d with different magnifications, Ni-CNTs evenly disperse on the surface of the prepared gas sensor. Hence, there will be large, hollow structures for gas diffusion among the Ni-CNTs, which not only increase the adsorption capacity, but reduce the adsorption and desorption time. Ni nanoparticles mainly distribute on the outside wall of CNTs due to the long length and closed structure of CNTs (about 30 μm). The small diameter of Ni-CNTs (about 20 nm) signifies large curvature and high surface activity. Besides, the surface defects that existed on the surface of CNTs provide the adsorption sites for gas molecules. As a result, Ni nanoparticles evenly decorate on the surface of CNTs with their size distinctly smaller than the diameter of CNTs. Ni nanoparticles act as the active sites for gas molecule adsorption, which effectively enhances the gas-sensing properties of CNT-based sensors.

**Figure 3:**
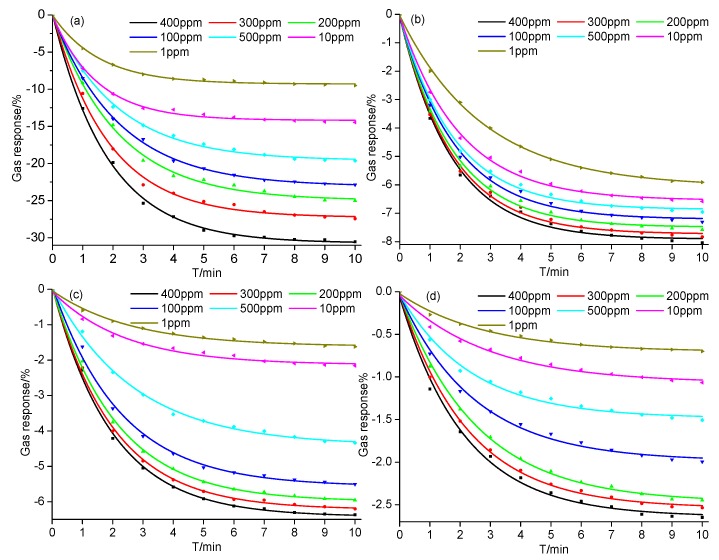
Gas response of the Ni-CNT gas sensor to different concentrations of SF_6_ decomposition components: (**a**) H_2_S, (**b**) SOF_2_, (**c**) SO_2_, and (**d**) SO_2_F_2_ at room temperature and ambient temperature.

Considering the concentration range of SF_6_ decomposition components generated in SF_6_-insulated equipment reported in previous studies [4,25], a series of SF_6_ decomposition components concentrations, 1, 10, 50, 100, 200, 300, and 400 ppm, were prepared to analyze the gas-sensing performance of the Ni-CNT gas sensor. As shown in Figure 3, the resistance of the gas sensor significantly reduces when it contacts SF_6_ decomposition components [26,27,28,29]. The Ni-CNT gas sensor presents a large gas response to SF_6_ decomposition components at room temperature and ambient pressure due to the catalytic property of evenly distributed Ni nanoparticles. Furthermore, the CNT sensor presents a fast gas response because of the large gas diffusion channel among Ni-CNTs, which also implies a fast gas-sensing recovery speed. The resistance rapidly reduces when the sensor just contacts the SF_6_ decomposition components, because the gas molecules quickly interact with the gas-sensing material on the surface of the Ni-CNT gas sensor. Meanwhile, the reduction speed gradually decreased as the gas diffusion rate sharply reduced with diffusion depth in the sensor film.

As shown in Figure 3, it takes about 10 min for the gas response to reach a steady state. The variation of gas response is greatly influenced by the concentration of SF_6_ decomposition components. For instance, the difference of gas response as the gas concentration increases from 300 to 400 ppm is smaller than that of 200 to 300 ppm. Comparing the value of gas responses shown in Figure 3a–d under the same gas concentration, the variation of gas responses is placed in the following order: H_2_S > SOF_2_ > SO_2_ > SO_2_F_2_. The largest value of gas response to H_2_S is −30.54% at the concentration of 400 ppm, and the value of the gas response still reached −9.50% at the lowest concentration (1 ppm) as shown in Figure 3a. The high gas response mainly comes from the strong interaction between Ni-CNTs and H_2_S molecules at the Ni atom functionalized sites, which contributes to the detection of the H_2_S component up to sub-ppm level. According to Figure 3b, the gas response of the Ni-CNT gas sensor to SOF_2_ changes a little under different gas concentrations. The gas responses at 400 ppm and 1 ppm are −8.04% and −5.91%, respectively. On the contrary, different concentrations of SO_2_ leads to a significant change of gas response, as shown in Figure 3c. The value of gas response increases from −1.62% (1 ppm) to −6.37% (400 ppm). Although Ni atom functionalization on the Ni-CNT surface enhances its adsorption to SO_2_F_2_, the Ni-CNT gas sensor was still not sensitive to SO_2_F_2_. As shown in Figure 3d, the gas responses to SO_2_F_2_ are −2.65% and −0.7% at 400 ppm and 1 ppm, respectively. In summary, the Ni-CNT gas sensor shows a high gas response to SF_6_ decomposition components, and its high gas-sensing selectivity can be used to identify the gas types of SF_6_ decomposition components produced in SF_6_-insulated equipment, making it suitable to detect the insulation defects and diagnose the running status of SF_6_ insulated equipment online.

Table 1 shows the comparison of the limit of detection (LOD) for different sensors to SF_6_ decomposition components reported in recent studies, including the most studied gas-sensing material: CNTs, graphene, and TiO_2_ nanotubes. In this study, the highest gas response of the Ni-CNT sensor studied in this work reaches −9.5% upon 1 ppm H_2_S measurement, which was slightly higher than that of reported CNT-based gas sensors. Meanwhile, a TiO_2_-based gas sensor usually needs a high working temperature to receive a high gas response to SF_6_ decomposition components. It was reported that the LOD of Au-Graphene was only 50 ppm with a gas response of 18.75% to H_2_S [28]. Therefore, the Ni-CNT sensor obviously presents advantages in high gas response and low working temperature.

**Figure 4:**
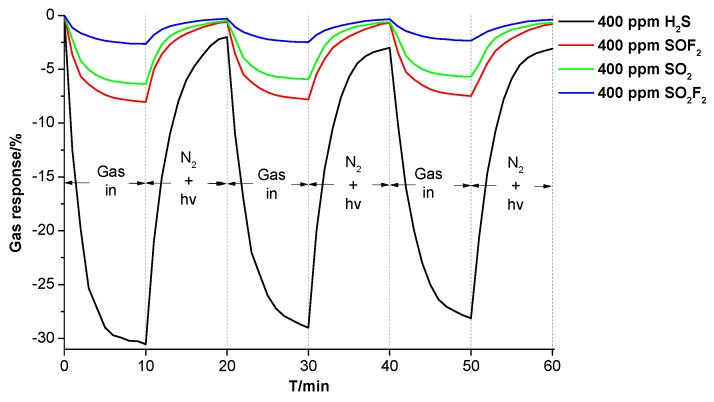
The gas response and recovery properties of the Ni-CNT gas sensor to SF_6_ decomposition components.

To demonstrate the reusability of the prepared Ni-CNT gas sensor, we have done multiple measurements to obtain its response and recovery properties to different types of SF_6_ decomposition components at the same concentration (400 ppm). As shown in Figure 4, the flow rate of SF_6_ decomposition components is 100 sccm during the gas-sensing process, and the N_2_ flow is used in the recovery process with a flow rate of 200 sccm. In addition, the illumination of UV light is applied to enhance the recovery process as it greatly increases the molecular vibration of SF_6_ decomposition components. Gas-in and N_2_ + UV represent the flow of SF_6_ decomposition components (SO_2_, H_2_S, SOF_2_, and SO_2_F_2_) and the flow of pure N_2_ flow with UV illumination, respectively. Simultaneously, N_2_ flow transfers the desorbed gas from the surface of Ni-CNTs and avoids re-adsorption. The Ni-CNT gas sensor still keeps a good gas response to SO_2_, H_2_S, SOF_2_, and SO_2_F_2_ after multiple gas adsorption and desorption detections, as the gas response changes little with measurement times. The value of gas response reduces from initially −30.54% to −28.13% upon H_2_S detection. In addition, the change of gas response to SOF_2_, SO_2_, and SO_2_F_2_ was 0.55% (from −8.04% to −7.49%), 0.69% (from −6.37% to 5.68%), and 0.31% (from −2.65% to −2.34%) after three lots of measurements, which is much less than the change of gas response to H_2_S.

**Figure 5:**
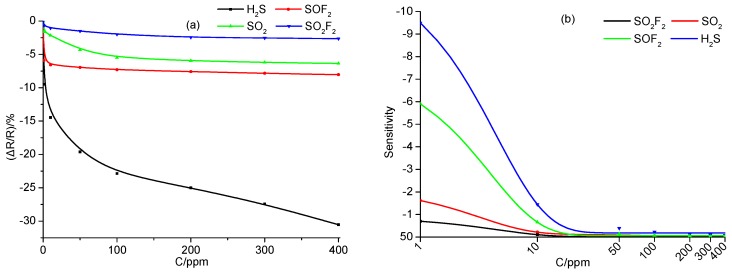
(**a**) The change of gas response, and (**b**) the change of sensitivity with the increase of concentration of SF_6_ decomposition components.

Figure 5a shows the change of gas response with the increase of concentration of SF_6_ decomposition components. Upon H_2_S and SOF_2_ detection, the gas response sharply enhances when gas concentration increases from 1 to 50 ppm, and then tends to linearly increase under high gas concentration of detected gases, from 50 to 400 ppm. This also demonstrates that the Ni-CNT sensor is sensitive to H_2_S and SOF_2_. Upon SO_2_ and SO_2_F_2_ detection, the increase of the gas response shows an exponential growth trend. Figure 5b shows the change of sensitivity to SF_6_ decomposition components; the sensitivity presents an exponential attenuation from 1 to 400 ppm, because it tends to be saturated at a high gas concentration. The attenuation speed is listed in the following order: H_2_S > SOF_2_ > SO_2_ > SO_2_F_2_ at the same gas concentration.

### 3.2. DFT Study of the Ni-CNT Gas Sensor

**Figure 6:**
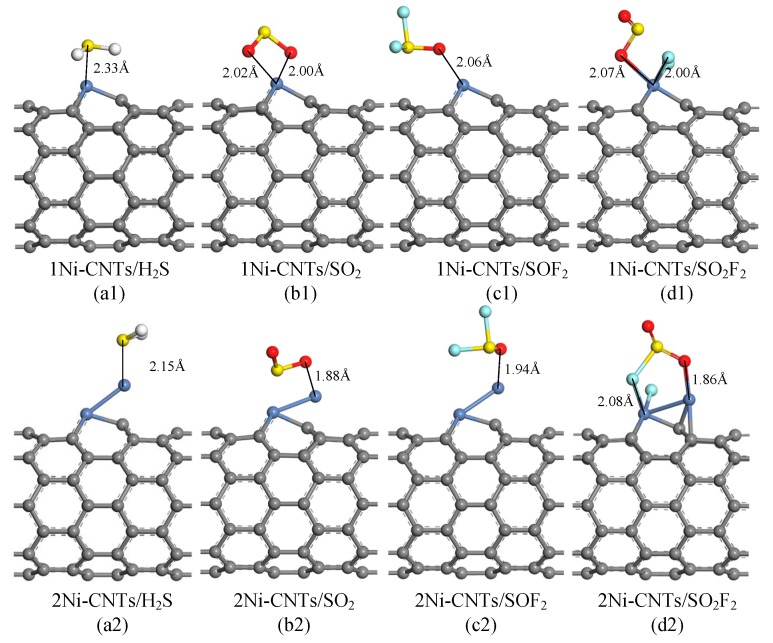
Optimized geometries of SF_6_ decomposition components adsorbed Ni-doped CNTs, distances in Å.

DFT study is used to analyze the adsorption properties of Ni-CNTs to SF_6_ decomposition components: SO_2_, H_2_S, SOF_2_, and SO_2_F_2_. As Ni nanoparticles modified on the CNT surface always exist as clusters, single and double Ni atom-doped CNT structures are built to reflect the influence of Ni nanoparticles, represented by 1Ni-CNTs and 2Ni-CNTs. Figure 6 and Table 2 show the most stable adsorption structures and corresponding data for one gas molecule adsorption on Ni-CNTs. It is found that all the gas molecules tend to adsorb around Ni atoms due to its high adsorption activity. SO_2_, H_2_S, and SOF_2_ interact with Ni-CNTs with physisorption. Due to the polyvalent properties of the sulfur atom, the chemisorption between SO_2_F_2_ and the Ni-CNT surface leads to the structure break of SO_2_F_2_ in the adsorption process, reducing the reusability of the CNT gas sensor as it impedes the gas molecule desorption. The H_2_S molecule interacts with Ni-CNTs by sulfur atoms. The nearest adsorption distance for H_2_S reduces from 2.33 Å to 2.15 Å when the doped Ni atoms increase from single to double. SO_2_ and SOF_2_ molecules interact with Ni-CNTs by oxygen atoms rather than sulfur atoms because the sulfur atom has already built a strong covalent bond with two oxygen atoms or two fluorine atoms. The nearest adsorption distance for SO_2_ and SOF_2_ on 2Ni-CNTs are 1.88 Å and 1.94 Å, respectively. The nearest distance from the SO_2_F that breaks from SO_2_F_2_ to Ni-CNTs is 1.86 Å on 2Ni-CNTs. Except for H_2_S adsorption, electrons transfer from Ni-CNTs to gas molecules in the adsorption process as Ni atoms act as the electron donor. The large adsorption energy for all of the gas molecules shows that Ni-doped CNTs are an effective gas-sensing material to detect the SF_6_ decomposition components.

**Figure 7:**
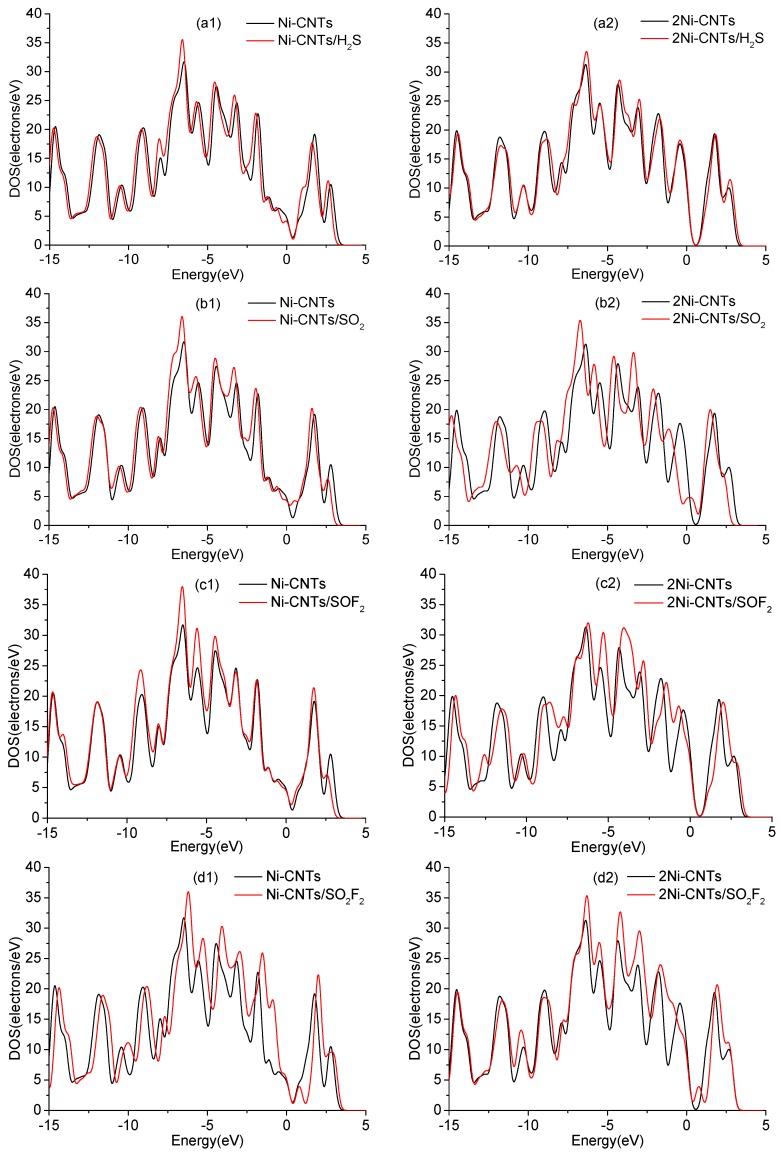
Density of states (DOS) of Ni-CNTs, 2Ni-CNTs, and gas adsorbed Ni-CNTs: (**a1**–**d1**) represent gas adsorption on Ni-CNTs, (**a2**–**d2**) represent gas adsorption on 2Ni-CNTs.

Generally, gas sensors detect the concentration of contacted gas by measuring the change of conductivity during the adsorption process. Density of states (DOS) is one of the effective ways to analyze the change of conductivity upon SO_2_, H_2_S, SOF_2_, and SO_2_F_2_ adsorption. As shown in Figure 7, the DOS around Fermi level for H_2_S, H_2_S, and SOF_2_ adsorptions obviously increase in different degrees, which leads to the corresponding change of conductivity. The change of conductivity has just verified that the Ni-CNT sensor shows a high gas response to H_2_S, H_2_S, and SOF_2_. Meanwhile, the inconspicuous change of DOS upon SO_2_F_2_ adsorption slightly increases the conductivity of the adsorption system, which is consistent with the experimental study.

In DOS analysis, the change of conductivity upon H_2_S adsorption is less than SO_2_, SOF_2_, and SO_2_F_2_ adsorption, based on the calculation condition that the default temperature is 0 K. However, the detection temperature has increased to ambient temperature, which distinctly enhances the reaction activity of the S atom. In addition, S in H_2_S is much more active than that in SO_2_, SOF_2_, and SO_2_F_2_ because it only builds two monovalences with H atoms. As a result, the change of conductivity for a single H_2_S molecule is the largest among four gas molecule adsorptions in the experiment.

## 4. Conclusions

This study introduces Ni modification on the CNT surface for the sensitive and selective detection of SF_6_ decomposition components: SO_2_, H_2_S, SOF_2_, and SO_2_F_2_. The TEM results show that Ni particles evenly distribute on the surface of CNTs. The prepared Ni-CNT gas sensor shows high gas sensitivity as its LOD reaches 1 ppm to SF_6_ decomposition components. The sensitivity of the Ni-CNT gas sensor is placed in the following order: H_2_S > SOF_2_ > SO_2_ > SO_2_F_2_. In addition, the Ni-CNT gas sensor shows good gas-sensing recovery property, which makes it easy to be widely used. DFT theoretical study results verify that conductivity of the adsorption system significantly changes when SF_6_ decomposition components interact with Ni-CNTs. Thus, our finding is certainly beneficial to broaden the perspective of Ni-CNT gas sensor applications in evaluating and diagnosing the insulation status of SF_6_-insulated equipment online.

## Figures and Tables

**Table 1:** The limit of detection (LOD) of different gas sensors to SF_6_ decomposition components. RT represents room temperature. CNTs: carbon nanotubes.

Sensor	LOD (ppm)	T (°C)	Gas	Reference
Ni-CNTs	1	RT	H_2_S	This work
Sieve/CNTs	10	RT	H_2_S	Zhang et al. [30]
Pd-CNTs	50	RT	H_2_S	Star et al. [31]
Pt-TiO_2_NTs	25	160	SO_2_	Jing et al. [32]
Au-Graphene	50	RT	H_2_S	Yu et al. [28]

**Table 2:** Interaction distance (*d*), adsorption energy *E*_ads_, charge transfer *Q*_t_ of SF_6_ decomposition components adsorbed Ni-doped CNTs.

System	*d* (Å)	*Q*_t_ (e)	*E*_ads_ (eV)
1Ni-CNTs/H_2_S	2.33	0.25	−0.81
1Ni-CNTs/SO_2_	2.00	−0.35	−1.13
1Ni-CNTs/SOF_2_	2.06	−0.06	−0.49
1Ni-CNTs/SO_2_F_2_	2.07	−0.94	−1.93
2Ni-CNTs/H_2_S	2.15	0.19	−3.05
2Ni-CNTs/SO_2_	1.88	−0.30	−2.26
2Ni-CNTs/SOF_2_	1.94	−0.26	−2.83
2Ni-CNTs/SO_2_F_2_	1.86	−0.98	0.97
